# Mitochondrial Transplantation Attenuates Neural Damage and Improves Locomotor Function After Traumatic Spinal Cord Injury in Rats

**DOI:** 10.3389/fnins.2022.800883

**Published:** 2022-04-12

**Authors:** Ming-Wei Lin, Shih-Yuan Fang, Jung-Yu C. Hsu, Chih-Yuan Huang, Po-Hsuan Lee, Chi-Chen Huang, Hui-Fang Chen, Chen-Fuh Lam, Jung-Shun Lee

**Affiliations:** ^1^Department of Medical Research, E-Da Hospital, E-Da Cancer Hospital, Kaohsiung City, Taiwan; ^2^Department of Nursing, College of Medicine, I-Shou University, Kaohsiung City, Taiwan; ^3^Regenerative Medicine and Cell Therapy Research Center, Kaohsiung Medical University, Kaohsiung City, Taiwan; ^4^Department of Anesthesiology, College of Medicine, National Cheng Kung University Hospital, National Cheng Kung University, Tainan City, Taiwan; ^5^Department of Cell Biology and Anatomy, College of Medicine, National Cheng Kung University, Tainan City, Taiwan; ^6^Institute of Basic Medical Sciences, College of Medicine, National Cheng Kung University, Tainan City, Taiwan; ^7^Section of Neurosurgery, Department of Surgery, College of Medicine, National Cheng Kung University Hospital, National Cheng Kung University, Tainan City, Taiwan; ^8^Department of Anesthesiology, E-Da Hospital, E-Da Cancer Hospital, Kaohsiung City, Taiwan; ^9^College of Medicine, I-Shou University, Kaohsiung City, Taiwan

**Keywords:** allogenic mitochondria, mitochondrial dysfunction, mitochondrial transplantation, oxidative stress, spinal cord injury

## Abstract

Mitochondrial dysfunction is a hallmark of secondary neuroinflammatory responses and neuronal death in spinal cord injury (SCI). Even though mitochondria-based therapy is an attractive therapeutic option for SCI, the efficacy of transplantation of allogeneic mitochondria in the treatment of SCI remains unclear. Herein, we determined the therapeutic effects of mitochondrial transplantation in the traumatic SCI rats. Compressive SCI was induced by applying an aneurysm clip on the T10 spinal cord of rats. A 100-μg bolus of soleus-derived allogeneic mitochondria labeled with fluorescent tracker was transplanted into the injured spinal cords. The results showed that the transplanted mitochondria were detectable in the injured spinal cord up to 28 days after treatment. The rats which received mitochondrial transplantation exhibited better recovery of locomotor and sensory functions than those who did not. Both the expression of dynamin-related protein 1 and severity of demyelination in the injured cord were reduced in the mitochondrial transplanted groups. Mitochondrial transplantation also alleviated SCI-induced cellular apoptosis and inflammation responses. These findings suggest that transplantation of allogeneic mitochondria at the early stage of SCI reduces mitochondrial fragmentation, neuroapoptosis, neuroinflammation, and generation of oxidative stress, thus leading to improved functional recovery following traumatic SCI.

## Introduction

Traumatic spinal cord injury (SCI) often leads to devastating neural consequences, including partial or total paralysis (Mothe and Tator, 2012). The pathophysiology of traumatic SCI is initiated by a mechanical injury, followed by a series of neuroinflammatory events that result in secondary injuries. Increased expression of proinflammatory cytokines, ionic imbalance, mitochondrial dysfunctions, overproduction of oxygen and nitrogen radicals, and subsequent necrotic and apoptotic cell death are major features of the secondary injuries (Hausmann, 2003; Pineau and Lacroix, 2007). Considering the impact of profound neuroinflammation on the outcomes of traumatic SCI, numerous clinical trials have been conducted aiming to alleviate neuroinflammation; however, none of these approaches have been successful. This consequence could be attributed to the limitations imposed by the complicated spatiotemporal course of neuroinflammation in SCI (Hurlbert et al., 2015; Devaux et al., 2016; Colon et al., 2018), which complicates choosing a proper time window for treatment. Hence, exploring a novel strategy that effectively counteracts inflammation-induced secondary injuries is critical for treating traumatic SCI. Recently, a mitochondria-targeted treatment has emerged as a potential anti-inflammatory intervention (Wang et al., 2019; Lee et al., 2021).

Healthy mitochondria—which function as a powerhouse of cells—are essential for cell survival. They govern energy production, which is released in the form of adenosine triphosphate (ATP). Mitochondrial dysfunction promotes the overproduction of reactive oxygen and nitrogen species (RONS) and perturbs intracellular calcium homeostasis (Ide et al., 2001; Bolanos et al., 2009; McEwen et al., 2011; Ahuja et al., 2017) and is a common pathway preceding cell death and evident in several diseases (Swerdlow et al., 2010; Gollihue et al., 2018) especially neurological disorders-including ischemic stroke, epilepsy, and other neurodegenerative diseases (Wu et al., 2019), since the nervous system is enormously energy dependent. In the central nervous system, mitochondria are transportable between cells; neurons release damaged mitochondria to astrocytes either for disposal or recycling under normal conditions (Davis et al., 2014). These astrocytes can transfer viable mitochondria to the ischemic neurons during cerebral infarction to support both their viability and recovery (Hayakawa et al., 2016). Moreover, the neurorestorative effect of the transplantation of endothelial progenitor cells is primarily mediated through the release of the active extracellular mitochondria that derived from the progenitor cells in a rat model of ischemic stroke (Garbuzova-Davis et al., 2017; Russo et al., 2018). Furthermore, transferring exogenous mitochondria to the injured hippocampal neuronal cultures not only significantly increases neurite regrowth, but also restores their membrane potential (Chien et al., 2018). This transferable feature of mitochondria enlightens the innovation of mitochondrial transplantation (MT) treatments, which rapidly increases the mitochondrial density around the lesioned regions by direct administration of healthy mitochondria to replace the dysfunctional counterparts (McCully et al., 2017). The beneficial effects of MT on ischemia/reperfusion-induced myocardial injuries (McCully et al., 2009, 2017) have been proven in animal and human studies. However, studies testing the therapeutic effects of MT on SCI-induced functional impairments and neuroinflammation-related secondary insults are limited and the results remained conflicting.

Accordingly, to address this issue, we hypothesized that MT could alleviate SCI-induced functional and histological deficits and neuroinflammation-associated secondary injuries. To test this hypothesis, we made compressive traumatic SCI in rats by applying an aneurysm clip on their T10 spinal cord for 20 s. A 100 μg of soleus-derived allogeneic mitochondria labeled with fluorescent tracker, MitoTracker Deep Red FM (MTDR), was transplanted into the injured spinal cords of rats after the induction of traumatic SCI. The effects of MT on the SCI-induced impairments of somatosensory and locomotor functions were determined by performing somatosensory evoked potentials (SSEP) assay and Basso, Beattie, and Bresnahan (BBB) scoring, respectively. Luxol fast blue (LFB) staining was adopted to examine the degrees of demyelination in the injured spinal cord. The levels of neural apoptosis and neuroinflammation in the injured region were determined with Western blots, terminal deoxynucleotidyl transferase dUTP nick end labeling (TUNEL) assay, and enzyme-linked immunosorbent assay (ELISA).

## Materials and Methods

### Animals

All experimental procedures were approved by the Institutional Animal Care and Use Committee. Adult Sprague–Dawley rats (220–250 g) were purchased from BioLASCO (Taipei, Taiwan) and maintained in the institutional Laboratory Animal Center. The rats were housed under an 11-h light/13-h dark cycle (lights on at 7 A.M.) at a stable temperature (24 ± 1°C) and humidity in the facility. The rats were given free access to food and water.

Fifty-six adult rats were used in this study. Six rats served as donors of allogeneic mitochondria and 40 rats were employed as experimental subjects. We used 10 rats to investigate the spatiotemporal distribution and viability of the transplanted mitochondria. These 10 rats were sacrificed on postinjury day (PID) 1, 3, 7, 10, 14, and 28 according to the experimental design. The remaining rats were used to investigate the effects of MT on the parameters of interest. They were randomly divided into four groups, i.e., sham laminectomy + vehicle control group, sham laminectomy + MT group, SCI + vehicle control group (Vehicle), and SCI + MT group. The MT rats were administered allogeneic mitochondria suspended in 1x phosphate-buffered saline (PBS), while the Vehicle rats were injected with an equal volume of 1x PBS. The details of MT were described in the following sections.

### Allogeneic Mitochondria Isolation and Labeling

Allogeneic mitochondria were freshly isolated from the bilateral soleus muscles of healthy donor rats. The donor rat was placed in a prone position under deep anesthesia with 4–5% of isoflurane (Panion & BF Biotech Inc., Taipei, Taiwan). An incision was made at the midline of the dorsal lower limb from its ankle to popliteal fossa. The superficial connective tissues and the gastrocnemius muscles were dissected to expose the soleus. Then, the bilateral soleus muscles were excised, immersed in 1x PBS, cut into tiny pieces, and homogenized with mitochondrial isolation solution (Cat. #: 89801, Thermo Fisher Scientific, Waltham, MA, United States) by a glass tissue grinder. The homogenates were centrifuged at 700 × *g* for 10 min at 4°C and the supernatants containing mitochondria were collected and centrifuged at 3,000 × *g* for 15 min at 4°C. After discarding the supernatants, the pellets were washed with wash buffer and centrifuged at 12,000 × *g* for 5 min at 4°C twice. Finally, the washed pellets were resuspended in 1x PBS and then stained with MTDR (Cat. #: M22425, Thermo Fisher Scientific) at 37°C for 30 min. The labeled mitochondria obtained from different donor rats were washed, suspended in 1x PBS, pooled together, and ready for further use.

### Procedures of Induction of Traumatic Spinal Cord Injury and Mitochondrial Transplantation

The rats were anesthetized by an intraperitoneal injection (i.p.) of Zoletil^®^ 50 (40 mg/kg; Virbac, Carros, France), administrated with enrofloxacin (5 mg/kg, Bayer, Leverkusen, Germany) and placed in a prone position. A 2-cm dorsal longitudinal incision was made over the T9-T10 vertebrae, dissected the paraspinal muscles, removed the spinal processes of T9-T10, and performed a laminectomy of the T10 vertebra to expose the spinal cord. Then, a new aneurysm clip (Model: No: 07-940-02, Sugita, Mizuho Logistics, Chiba, Japan) was extramurally applied on the T10 spinal cord for 20 s to elicit compressive traumatic SCI.

Following SCI, we used a microsyringe pump (Model: KDS101, KD Scientific, Holliston, MA, United States) to transplant mitochondria into the injured region of spinal cord of rats via the intraparenchymal route. Each rat received two injections which were conducted at 2 mm rostral and caudal to the epicenter of the injured site (0.6 mm in depth), respectively. The MT rats received administrations of mitochondria (50 μg in 1.5 μL 1x PBS, each shot), and the Vehicle rats received injections of equal volume of 1x PBS and served as controls. Then, the wound was closed in layers. The rats recovered from anesthesia under a warm blanket, housed individually, and had free access to water and chow. The urinary bladder of each rat was manually voided twice daily until the recovery of the bladder reflex.

### Assessment of Sensory and Locomotor Functions of Rats

The sensory function of the hind limbs was evaluated using SSEP on PID 28 (Lee et al., 2012). The rats were anesthetized using Zoletil^®^ 50 (40 mg/kg, i.p.; Virbac, Carros, France) and kept in a prone position. A needle electrode was inserted into the plantar aspect of the foot to stimulate the tibial nerve with rectangular pulses at a supramaximal intensity of 7 Hz for a duration of 0.2 ms. The needle electrode recorder was inserted into the C2-C3 interspinous ligament. The reference electrode was placed in the subcutaneous tissue next to the recording electrode. In contrast, the ground electrode was placed in the shoulder, ipsilateral to the side being stimulated. The recorded signals were averaged 20–50 times at a band-pass filter setting of 50–5,000 Hz, with a 20-ms time base. During the whole process, the heart rate, blood pressure, and core temperature of the rats were monitored.

The hindlimb locomotor function was independently evaluated by two observers which were blinded to the treatment groups using the BBB scale weekly (Lee et al., 2020).

### Western Blotting

To collect spinal cord samples for Western blot, the rats were deeply anesthetized with Zoletil^®^ 50 (40 mg/kg, i.p.; Virbac, Carros, France) and transcardially perfused with chilled normal saline. The 1-cm sections of the spinal cords centered at the epicenter of the injured site were dissected and homogenized with T-PER lysis buffer (Cat. #: 78510, Thermo Fisher Scientific Inc.) containing protease inhibitors (Cat. #: 04693116001, Roche, Basel, Switzerland) and phosphatase inhibitors (PHOSS-RO, Roche). After centrifuging at 15,000 × *g* for 15 min, the supernatants (25 μg) were loaded onto polyacrylamide gels (9–12%), electrophoresed with Mini-PROTEAN^®^ tetra cell system (Bio-Rad Laboratories, Hercules, CA, United States), and transferred to PVDF membranes (Cat. #: IPVH00010, Merck-Millipore, Burlington, MA, United States) with wet transfer tank (Model: TE22 Mighty Small Transfer Tank, Hoefer, Holliston, MA, United States). The membranes were incubated overnight at 4°C with appropriate dilutions of primary antibodies, including cleaved caspase-3 (1:1,000; Cat. #: 9661, Cell Signaling Technology, Danvers, MA, United States), Bcl-2 (1:1,000; Cat. #: ab59348, Abcam, Cambridge, United Kingdom), BAX (1:1,000; Cat. #: 2772, Cell Signaling Technology), dynamin-related protein 1 (Drp1) (1:1,000; Cat. #: 8570, Cell Signaling Technology), tumor necrosis factor (TNF) (1:2,000, Cat. #: ab6671, Abcam), interleukin-6 (IL-6) (1:1,000, Cat. #: ab6672, Abcam), inducible nitric oxide synthase (iNOS) (1:1,000; Cat. #: A0312, ABclonal, Woburn, MA, United States), and β-actin (1:10,000; Cat. #: ab8227, Abcam). The band densities were measured using an image system (Model: Azure 280, Azure Biosystems, Dublin, CA, United States) and the densitometry was carried out using the ImageJ software (v2.0.0-rc-69/1.52p, U.S. National Institutes of Health). Relative protein expression was estimated by normalizing with the β-actin level. For re-probing, the membranes were incubated with a stripping buffer containing 2% SDS, 62.5 mM Tris, and 0.8% 2-mercaptoethanol for 20 min at 55°C to remove the bound antibodies.

### Measurement of Nitric Oxide, 3-Nitrotyrosine, and Malondialdehyde

We determined the tissue concentrations of nitric oxide (NO), 3-nitrotyrosine (3-NT), and malondialdehyde in the spinal cord homogenates, respectively, using the Griess reagent kit (Cat. #: G7921, Sigma-Aldrich, St. Louis, MO, United States), 3-NT ELISA kit (Cat. #: ab113848, Abcam), and malondialdehyde assay kit (Cat. #: NWK-MDA01, Northwest Life Science Specialties, Vancouver, WA, United States) according to the manufacturers’ protocols.

### Histological Examination

To determine the distribution of the transplanted mitochondria, the 1-cm sections of the spinal cords centered at the epicenter of the injured site were post-fixed with 4% paraformaldehyde and prepared into 20-μm thick horizontal sections using a cryostat. For the TUNEL assay and LFB staining, the spinal section was post-fixed with 4% paraformaldehyde and prepared into 20-μm thick transverse sections using a cryostat.

The TUNEL assay and LBF staining were carried out with commercial kits (TUNEL assay: Cat. #: S7165, Sigma-Aldrich; LFB staining: Cat. #: ab150675, Abcam). The labeled mitochondria and the TUNEL-positive nuclei were visualized under a fluorescence microscope (Nikon Microsystems). The LFB-stained sections were photographed at low magnification. Moreover, the ratio of the LFB-positive area to the total cross-sectional area of the spinal cord was quantified using ImageJ software and presented as a percentage. The images of the aforementioned experiments were captured with a fluorescence optical microscope (Model: ECLIPSE Ci, Nikon, Tokyo, Japan) equipped with a digital camera (Model: DS-Fi3, Nikon).

### Statistical Analysis

All data are presented as the mean ± standard deviation. All statistical analyses were conducted using the Prism 7th edition. Two-tailed Student’s *t*-test was used to compare means between two groups. The BBB scoring was analyzed by repeated-measures two-way ANOVA followed by Sidak’s multiple comparisons. Significance was set at *p* < 0.05.

## Results

### Spatiotemporal Distribution and Viability of the Transplanted Mitochondria in the Injured Spinal Cord

**FIGURE 1:**
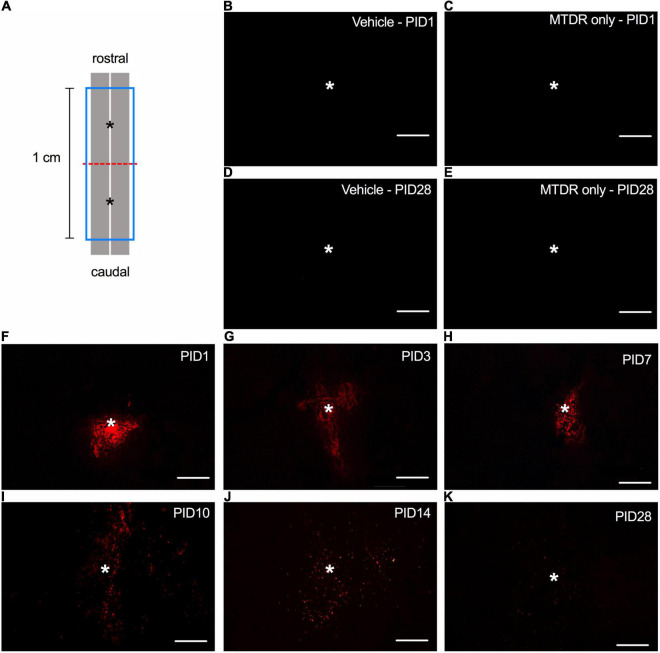
The spatiotemporal distribution of transplanted mitochondria in the injured spinal cord of rats with traumatic SCI. **(A)** Diagram of the schematic spinal cord. The red dash line indicated the injury site. The blue frame indicated the 1-cm sampling area. The asterisks denoted the sites of administration of allogeneic mitochondria. **(B)** Representative micrograph of MTDR signals in the injured spinal cord of rat injected with 1x PBS vehicle on PID 1. **(C)** Representative micrograph of MTDR signals in the injured spinal cord of rat injected with MTDR dye on PID 1. **(D)** Representative micrograph of MTDR signals in the injured spinal cord of rat injected with 1x PBS vehicle on PID 28. **(E)** Representative micrograph of MTDR signals in the injured spinal cord of rat injected with MTDR dye on PID 28. **(F–K)** Representative micrograph of MTDR signals which were detected on PID1, 3, 7, 10, 14, and 28 in the injured spinal cord of rats injected with MTDR-labeled mitochondria. Horizontal sections were used. Scale bar = 100 μm. *N* = 1 rat per panel. The asterisks indicate the points of injection.

The amount (50 μg × 2, resuspended in 1x PBS) of transplanted mitochondria administered in this presented study was similar to the effective dosage characterized by Gollihue et al. (2018). Initially, we detected the MTDR signals in the horizontal sections of the injured spinal cord of rats (Figure 1A) that received MT to demonstrate the spatiotemporal distribution of transplanted mitochondria. No MTDR signal was detected in the injured spinal cord of rats injected with vehicle (Figures 1B,D) or MTDR dye (Figures 1C,E) on PID 1 and 28, suggesting that there was no obvious confoundedness of autofluorescence and that MTDR stained the exogenous mitochondria only. In the sections of the spinal cord with MTDR-labeled mitochondria, the MTDR signals were detectable at time points of PID 1, 3, 7, 10, 14, and 28 (Figures 1F–K). The exogenous MTDR-labeled mitochondria appeared as a cluster on PID 1 (Figure 1F) and gradually spread to the rostral and caudal ends of the lesion (Figures 1G–K).

### Mitochondrial Transplantation Improves Recoveries of Sensory and Locomotor Functions in Rats With Traumatic Spinal Cord Injury

**FIGURE 2:**
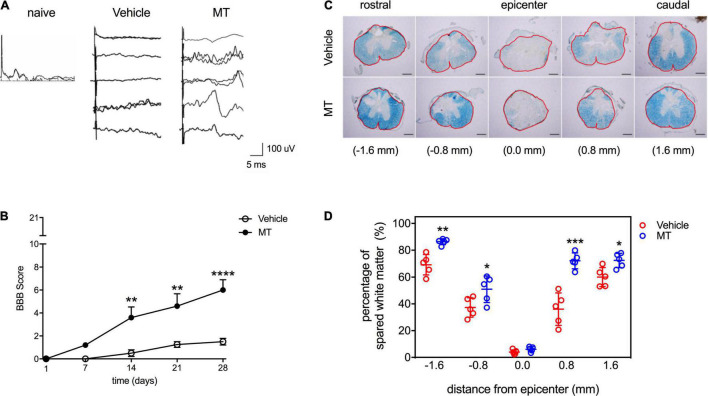
Effects of mitochondrial transplantation on the sensory and locomotor functions and the white matter sparing in the injured spinal cord of rats with traumatic SCI. **(A)** Representative micrographs of SSEP traces recorded in SCI rats on PID 28. Scale = 100 μV/5 ms **(B)** Quantitative results of BBB scoring. **p < 0.01, ****p < 0.0001, Sidak’s multiple comparisons performed after the repeated measures two-way ANOVA. **(C)** Representative micrographs of LFB staining of transverse sections prepared from the injured spinal cords of rats. Scale bar = 100 μm. The red frames indicated the analyzed areas. **(D)** Quantitative results of percentage of LFB-positive area. *p < 0.05, **p < 0.01, ***p < 0.001, two-tailed Student’s *t*-test. Data were expressed as mean ± standard deviation. *N* = 5. MT: mitochondria trans-plantation.

The effects of MT on the sensory function of traumatic SCI rats were examined by SSEP assessment performed on PID 28. Our results showed that there were no discriminable waveforms in the Vehicle group, whereas SSEPs were recognized in four out of five MT rats (Figure 2A). The locomotor functions of SCI rats were scored with the BBB test. Complete paralysis of the hind limbs in both groups was noted (BBB = 0) on PID 1 (Figure 2B). The MT rats showed better recovery of hindlimb locomotor function than the Vehicle ones in the BBB test carried out on PID 14 (3.60 ± 2.07 vs. 0.50 ± 0.58, score, MT vs. Vehicle, *p* < 0.01), 21 (4.60 ± 2.41 vs. 1.25 ± 0.50, score, MT vs. Vehicle, *p* < 0.01) and 28 (6.00 ± 2.00 vs. 1.50 ± 0.58, score, MT vs. Vehicle, *p* < 0.0001) (Figure 2B).

### Mitochondrial Transplantation Protects Against Traumatic Spinal Cord Injury-Induced Demyelination in the Injured Spinal Cord of Rats

The results of LFB staining revealed that the severity of demyelination increased as the distance from the epicenter of the injured site decreased in both Vehicle and MT rats (Figure 2C). MT improved the preservation of white matter in the injured region (−1.6 mm: 86.39 ± 2.20 vs. 69.26 ± 7.57%, MT vs. Vehicle, *p* < 0.01; −0.8 mm: 50.96 ± 9.91 vs. 37.30 ± 7.26%, MT vs. Vehicle, *p* < 0.05; + 0.8 mm: 72.09 ± 5.89 vs. 36.07 ± 12.11%, MT vs. Vehicle, *p* < 0.001; + 1.6 mm: 72.39 ± 5.25 vs. 60.05 ± 7.10%, MT vs. Vehicle, *p* < 0.05) of spinal cords (Figures 2C,D).

### Mitochondrial Transplantation Ameliorates Mitochondrial Fragmentation and Cellular Apoptosis in the Injured Spinal Cord of Rats With Traumatic Spinal Cord Injury

**FIGURE 3:**
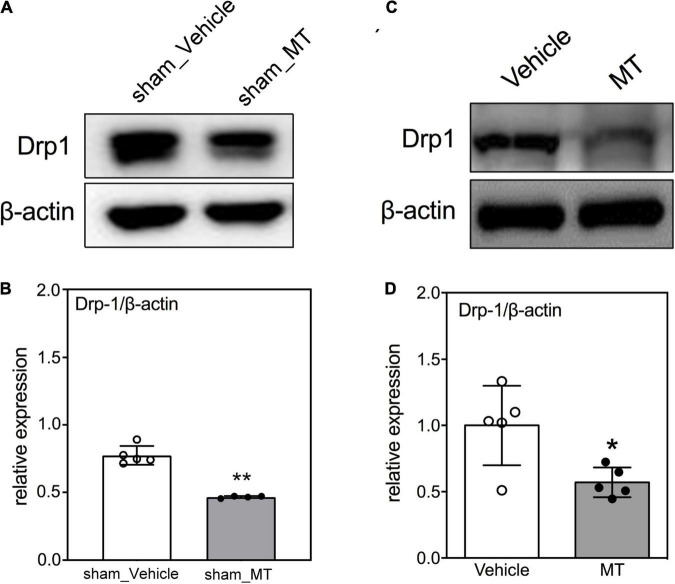
Effects of mitochondrial transplantation on Drp1 expression in the injured spinal cord of rats with traumatic SCI on PID 1. **(A)** Representative micrograph of Western blot analysis of Drp1 in sham control groups. **(B)** Corresponding quantitative results of relative expression of Drp1 in sham control groups. **(C)** Representative micrograph of Western blot analysis of Drp1 in SCI groups on PID 1. **(D)** Corresponding quantitative results of relative expression of Drp1 in SCI groups on PID 1. The 1-cm spinal cord specimens centered at the epicenter of the injured site were used in Western blots. Data were expressed as mean ± standard deviation. *p < 0.05, **p < 0.01, two-tailed Student’s *t*-test. *N* = 5. MT, mitochondria transplantation.

The mitochondrial dynamics of fusion and fission are crucial for mitochondrial homeostasis. It has been reported that mitochondrial fission occurs during early apoptosis and mitochondrial fragmentation-related mitochondrial dysfunction is linked to subsequent cell death (Liu et al., 2015; Jia et al., 2016). Accordingly, Western blotting was used to determine the protein level of Drp1, a marker of mitochondrial fission and fragmentation, in the injured sham control or injured spinal cords and found that MT rats had a lower Drp1 level (0.45 ± 0.12 vs. 1.00 ± 0.24, relative expression, MT vs. Vehicle, *p* < 0.01) than the Vehicle rats in the SCI groups on PID 1 (Figure 3).

**FIGURE 4:**
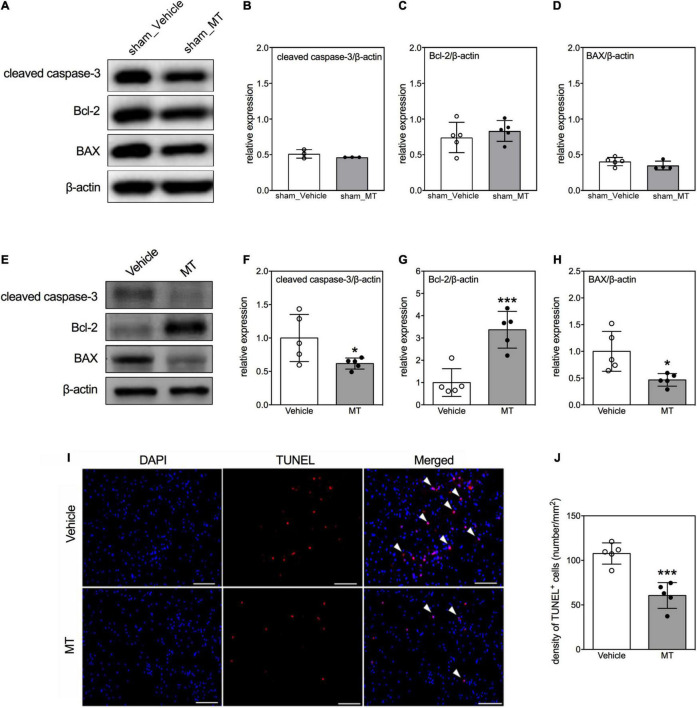
Effects of mitochondrial transplantation on cellular apoptosis in the injured spinal cord of rats with traumatic SCI. **(A)** Representative micrograph of Western blots of cleaved caspase-3, Bcl-2, and BAX in sham control groups. **(B–D)** Corresponding quantitative results of relative expression of cleaved caspase-3, Bcl-2, and BAX in sham control groups. **(E)** Representative micrograph of Western blots of cleaved caspase-3, Bcl-2, and BAX in SCI groups on PID 1. **(F–H)** Corresponding quantitative results of relative expression of cleaved caspase-3, Bcl-2, and BAX in SCI groups on PID 1. The 1-cm spinal cord specimens centered at the epicenter of the injured site were used in Western blots. **(I)** Representative micrographs of TUNEL assay. The white arrowheads indicate both DAPI and TUNEL dual-positive cells. The sections used in TUNEL assay were obtained from the 1-cm spinal cord specimens centered at the epicenter of the injured site. Scale = 100 μm. **(J)** Quantitative results of the TUNEL assay. Data were expressed as mean ± standard deviation. *p < 0.05, ***p < 0.001 two-tailed Student’s *t*-test. *N* = 3–5. MT, mitochondria transplantation.

Furthermore, we examined the effects of MT on cellular apoptosis in the injured spinal cord as well. First, we adopted immunoblotting to examine the expression of apoptosis regulatory proteins, i.e., cleaved caspase-3, Bcl-2, and BAX, in the regions of interest (Figures 4A–H) on PID 1. Quantitative results showed that apoptotic related proteins were not changed in sham control groups (Figures 4A–D). However, MT downregulated the expression of cleaved caspase-3 (0.68 ± 0.19 vs. 1.00 ± 0.18, relative expression, MT vs. Vehicle, *p* < 0.05) and BAX (0.71 ± 0.21 vs. 1.00 ± 0.07, relative expression, MT vs. Vehicle, *p* < 0.05) but upregulated the Bcl-2 (1.48 ± 0.27 vs. 1.00 ± 0.09, relative expression, MT vs. Vehicle, *p* < 0.01) level in the injured spinal cord of rats on PID 1 (Figures 4E–H). Second, TUNEL assay was performed to visualize the population of apoptotic cells in the injured spinal cord on PID 28. The results revealed that MT reduced the density of apoptotic cells (60.63 ± 14.46 vs. 107.74 ± 11.93, number/mm^2^, MT vs. Vehicle, *p* < 0.001) in the injured spinal cord (Figures 4I,J).

### Mitochondrial Transplantation Suppresses the Expression of Pro-inflammatory Cytokines in the Injured Spinal Cord of Rats With Traumatic Spinal Cord Injury

**FIGURE 5:**
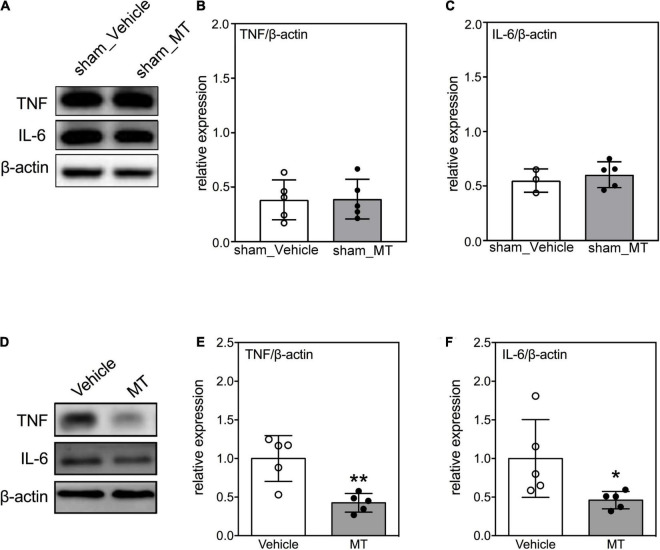
Effects of mitochondrial transplantation on expression of pro-inflammatory cytokines in the injured spinal cord of rats with traumatic SCI on PID 1. **(A)** Representative micrograph of Western blots of TNF and IL-6 in sham control groups. **(B,C)** Quantitative results of relative expression of TNF and IL-6 in sham control groups. **(D)** Representative micrograph of Western blots of TNF and IL-6 in SCI groups on PID 1. **(E,F)** Quantitative results of relative expression of TNF and IL-6 in SCI groups on PID 1. The 1-cm spinal cord specimens centered at the epicenter of the injured site were used in Western blots. Data were expressed as mean ± standard deviation. *p < 0.05, **p < 0.01, two-tailed Student’s *t*-test. *N* = 3–5. MT, mitochondria transplantation.

Neuroinflammation is accompanied by an increase of secretion of pro-inflammatory cytokines are critical hallmarks of secondary injury after SCI (Beattie et al., 2000; Bains and Hall, 2012). We confirmed that the inflammatory cytokines were not elevated after MT in sham control groups (Figures 5A–C). Moreover, we evaluated the levels of pro-inflammatory cytokines in the injured spinal cord after MT and demonstrated that MT rats had lower expression of TNF (0.57 ± 0.16 vs. 1.00 ± 0.10, relative expression, MT vs. Vehicle, *p* < 0.01) and IL-6 (0.49 ± 0.20 vs. 1.00 ± 0.14, relative expression, MT vs. Vehicle, *p* < 0.01) than the Vehicle control rats (Figures 5D–F).

### Mitochondrial Transplantation Attenuates Oxidative Damages in the Injured Spinal Cord of Rats With Traumatic Spinal Cord Injury

**FIGURE 6:**
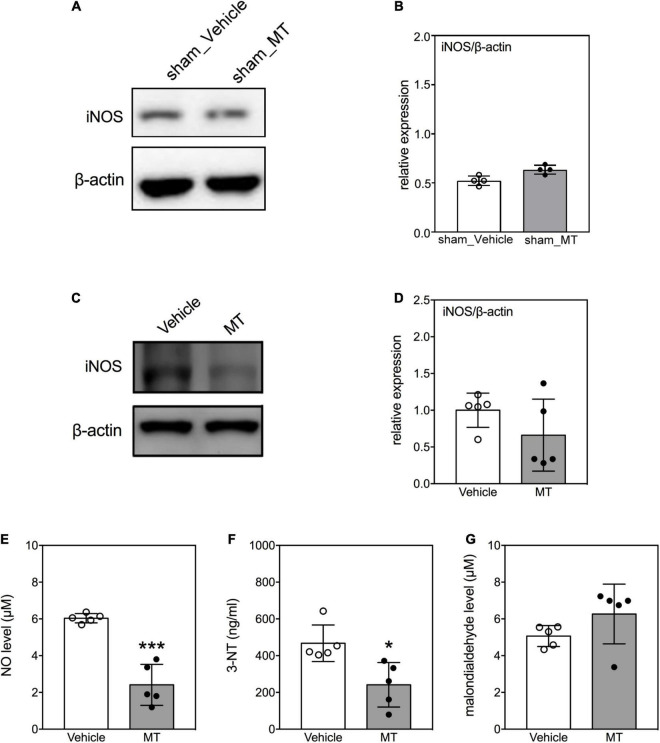
Effects of mitochondrial transplantation on oxidative stress in the injured spinal cord of rats with traumatic SCI on PID 1. **(A)** Representative micrograph of Western blot of iNOS in sham control groups. **(B)** Quantitative results of relative expression of iNOS in sham control groups. **(C)** Representative micrograph of Western blot of iNOS in SCI groups on PID 1. **(D)** Quantitative results of relative expression of iNOS in SCI groups on PID 1. The 1-cm spinal cord specimens centered at the epicenter of the injured site were used in Western blots. **(E–G)** Quantitative results of the level of NO, 3-NT and malondialdehyde in SCI groups. Data were expressed as mean ± standard deviation. *p < 0.05, ***p < 0.001, two-tailed Student’s *t*-test. *N* = 4–5. MT, mitochondria transplantation.

Overproduction of iNOS-derived NO and generation of 3-NT are signatures of oxidative damage during neuronal injury (Visavadiya et al., 2016). We investigated the effects of MT on the levels of these indicators of oxidative damage in the injured spinal cord on PID 1. The results demonstrated that the level of iNOS was not changed in the sham control groups (Figures 6A,B). In the injured groups, MT rats exhibited lower levels of iNOS (0.39 ± 0.06 vs. 1.00 ± 0.24, relative expression, MT vs. Vehicle, *p* < 0.01, Figures 6C,D), NO (2.41 ± 1.11 vs. 6.04 ± 0.25 μM, MT vs. Vehicle, *p* < 0.001, Figure 6E), and 3-NT (241.31 ± 121.06 vs. 467.33 ± 99.52 ng/ml, MT vs. Vehicle, *p* < 0.001, Figure 6F) than the Vehicle control rats on PID1. However, the concentrations of malondialdehyde, a marker for lipid peroxidation, were similar between the MT and Vehicle groups (Figure 6G).

## Discussion

This study was designed to elucidate the therapeutic effects of transplantation of allogeneic mitochondria on traumatic SCI. Our results showed that MT improved the recovery of somatosensory and locomotor functions after SCI and promoted the preservation of white matter in the injured spinal cord of rats with traumatic SCI. Attenuated mitochondrial fragmentation, cellular apoptosis, neuroinflammation, and oxidative stress were also evident in the spinal cord of rats that received MT. Moreover, by addressing the spatiotemporal distribution of the viable transplanted allogeneic mitochondria, we were able to newly demonstrate that transplanted allogeneic mitochondria survived in the lesioned region for at least 28 days in a rat model of traumatic SCI.

To the best of our knowledge, only two studies investigating the therapeutic effects of MT on traumatic SCI have been published (Li et al., 2015; Gollihue et al., 2018). Gollihue et al. (2018) first demonstrated that culture- and muscle-derived mitochondria, which were transplanted in the injured spinal cord, maintained their bioenergetics on PID 1. However, they did not identify any improving effect of MT on functional recovery in SCI rats (Gollihue et al., 2018). Conversely, Li et al. (2015) reported that the transplantation of mitochondria isolated from bone marrow mesenchymal stem cells to the injured spinal cord improved locomotor functional recovery in SCI rats, which is in line with our findings. These discrepancies may derive from the differences in the injecting site and the frequency of spinal cord puncture. First, intraparenchymal injection inevitably causes SCI. The midline of the dorsal spinal cord, the posterior median sulcus, has relatively fewer neural networks, compared with other parts of the spinal cord (Jacquesson et al., 2014; Mii et al., 2021). The midline incision is of paramount importance during surgery for intramedullary spinal pathologies. This can be attributed to its role in minimizing neural damage. Second, reduction in puncture time can mitigate tissue trauma. Therefore, we used a midline injection with two injection attempts, namely, rostral and caudal to the epicenter (Li et al., 2019), rather than a circumferential injection with four injection attempts (Gollihue et al., 2018).

It has been reported that transplanted mitochondria maintain their bioenergetics after transplantation (Garbuzova-Davis et al., 2017; Chien et al., 2018; Gollihue et al., 2018; Li et al., 2019); however, few studies have addressed their viability in the host. MTDR and mitochondria-targeting transgenically labeled green fluorescent protein (tGFP) are two widely used tools to label and trace the transplanted mitochondria. Given that the fluorescent signal of mitochondria-targeting tGFP decayed over time (Gollihue et al., 2018), we adopted MTDR to label the allogeneic transplanted mitochondria in this study. MTDR, a mitochondrial potential-dependent dye, stains viable mitochondria and sustains along with their viability (Xiao et al., 2016). Hence, MTDR is also suitable for detecting the viability of transplanted mitochondria. We found that the MTDR signals could be recognized in the injured spinal cord on PID 28, suggesting that the transplanted mitochondria survived for a least 28 days in the lesioned region. Our results were in line with a previous finding that transplanted xenogeneic mitochondria survived for 4 weeks in the myocardium of pigs with cardiac ischemia (Kaza et al., 2017). Furthermore, by characterizing the spatial distribution of the transplanted mitochondria, we also found that the spread region of the transplanted mitochondria was relatively restricted around the injection site. Hence, multiple and repeated administrations of allogeneic mitochondria may be required when dealing with a large volume of damaged tissue.

Intact mitochondrial function and bioenergetics depend on balanced mitochondrial fusion and fission. This mitochondrial dynamic is reactive to environmental changes. SCI leads to a shift from fusion to fission and subsequently results in neural death (Liu et al., 2015). It has been reported that the expression of mitochondrial fusion protein was increased, whereas the level of mitochondrial fission protein decreased in the spinal cord at 8 h after acute SCI (Jia et al., 2016). The expression of the mitochondrial fusion protein was downregulated, but the level of mitochondrial fission protein was upregulated at 24 h after acute SCI (Jia et al., 2016). Herein, we assessed mitochondrial fission by determining Drp1 expression and found that MT suppressed the expression of Drp1 in the spinal cord of SCI rats, suggesting that MT alleviates injury-induced mitochondrial fission. Our results are consistent with reports of pharmacological inhibition of mitochondrial fission improving locomotor functions in several SCI models (Li et al., 2015; Liu et al., 2015). The ratio of fusion/fission protein expression was suggested to be an important factor for maintenance of neuronal mitochondrial morphology and viability (Uo et al., 2009). We evaluated the ratio of Drp1/Mfn1 between the PBS and MT in the Sham and SCI groups. The results demonstrated that the ratio of Drp1/Mfn1 was decreased after MT in SCI group (Supplementary Figure 1). Our results were suggested the neuroprotection effects after MT in SCI rats.

Apoptosis, one of the leading causes of neural death in SCI, propagates from the injured site into the periphery and lasts for several weeks (Springer et al., 1999; Beattie et al., 2000). Inhibiting apoptosis improves locomotor functions in SCI models (Pei et al., 2017; Wei et al., 2018). Our results showed that MT repressed traumatic SCI-induced apoptosis by increasing the expression of anti-apoptotic protein, Bcl-2, and reducing the level of pro-apoptotic protein, BAX, the expression of apoptotic marker, cleaved caspase-3, and the TUNEL-positive cells. In line with our results, MT also exerted an anti-apoptotic effect in the model of cardiac ischemia (McCully et al., 2009, 2017). In SCI, apoptosis has been linked to mitochondrial fission according to the finding that the Drp1 inhibitor ameliorated SCI-induced apoptosis (Li et al., 2015). Thus, mitochondrial dynamics may be an upstream factor regulating the subsequent apoptosis. This relationship between mitochondrial fission and apoptosis was also evident in this study.

Mitochondria govern the homeostasis of cellular oxidative stress. An imbalance between the production and clearance of free radicals causes overwhelming injury to cells (Lobo et al., 2010). Since the mitochondrial content is relatively high in neurons, mitochondrial dysfunction following SCI elicits a huge accumulation of free radicals (Sullivan et al., 2007; Zhang et al., 2018). Antioxidant therapies are known to exert neuroprotective effects against SCI (Bains and Hall, 2012; Yang et al., 2016). In this study, we found that MT reduced the levels of NO, iNOS, and 3-NT in the injured spinal cord of rats. NO and iNOS are activators for the generation of RONS. 3-NT is a marker of protein oxidation in the injured spinal cord. Taken together, antioxidation may be involved in the mechanisms underlying the therapeutic effects of MT on traumatic SCI.

Activated microglia and infiltrating immune cells release a remarkable amount of pro-inflammatory mediators, such as TNF, IL-6, and NO, within hours after SCI and subsequently initiate a catastrophic secondary injury (Carlson et al., 1998; Hausmann, 2003; Pineau and Lacroix, 2007). Inhibiting the surge of inflammatory cytokines improves the functional outcomes in rats with SCI (Coelho-Santos et al., 2012; Guerrero et al., 2012; Wei et al., 2018). Herein, we observed a reduction in the TNF, IL-6, and NO levels in the injured spinal cords of MT rats, indicating reduction in inflammatory markers of MT. Similar to our findings, MT-induced downregulation of TNF and IL-6 has been also identified in a rabbit model of myocardial ischemia (Masuzawa et al., 2013). Our results suggested that anti-inflammation may be involved in the mechanisms underlying the therapeutic effects of MT on traumatic SCI. Moreover, it is noteworthy that IL-6 also behaves as a neurotrophic factor (Wagner, 1996; Erta et al., 2012) and promotes neuronal axon regeneration (Leibinger et al., 2013). Hence, the role of IL-6 in the MT-induced beneficial effects on SCI deserves further investigation.

Sirtuin 3 (SIRT3) has been identified as a stress-responsive deacetylase (Lombard et al., 2007), which shown to play a role in protecting cells under stress in the mitochondria (Bause and Haigis, 2013). It can exhibit mighty anti-inflammation and anti-oxidation upon neuronal injury (Huang et al., 2019; Ye et al., 2019). Mitochondrial SIRT3 is known to act as a pro-survival factor, playing an essential role to protect neurons under excitotoxicity (Kim et al., 2011). Dai et al. show that SIRT3 attenuates oxidative stress-induced mitochondrial dysfunction via coordination of mitochondrial biogenesis and fission/fusion (Dai et al., 2014). Overexpression or activation of SIRT3 can provide an incremental protective effect against oxidative injury (Pillai et al., 2010; Dai et al., 2014). Although there was no significant change in anti-inflammatory markers, our results showed the increased expression of SIRT3 after MT in the SCI group (Supplementary Figure 2), which suggested that MT ameliorated SCI-induced neuronal injury may relate to the SIRT3-mediated anti-inflammatory pathway.

Previous *in vivo* and *in vitro* studies have reported evidence of intracellular transmission of mitochondria under normal physiological and pathological conditions via tunneling nanotubes, extracellular vesicles, cellular fusion, and gap junctions (Torralba et al., 2016). However, evidence of intercellular transmission of exogenous mitochondria following mitochondrial transplantation is still undetermined. Very limited number of exogenous mitochondria were internalized into the host cells in experimental models of spinal cord and myocardial injury (McCully et al., 2009; Li et al., 2019). Similarly, our results also showed that the regional administration of viable allogenic mitochondria dispersed into the periphery, where most of these exogenous organelles were located interstitially, rather than being internalized into the recipient cells (Supplementary Figure 3). Furthermore, the transplanted mitochondria have been hypothesized to increase ATP generation in the mitochondrial dysfunction tissues in order to support the injured cells. However, this hypothesis was not supported by the fact that administration of mitochondrial components or ATP/adenosine diphosphate failed to reproduce the therapeutic effects of MT on ischemic myocardial injury (McCully et al., 2009). On the other hand, recent reports showed that transplanted mitochondria suppressed the micro-environmental Ca^2+^ overload through the Ca^2+^-buffering capacity to protect the neighboring cells (Chang et al., 2019). Additionally, Al Amir Dache et al. (2020) show human blood contains circulating cell-free respiratory competent mitochondria, which is suggestive of the maintenance of their bioenergetics under physiological calcium concentrations. However, the exact contribution of Ca^2+^-buffering capacity of MT on attenuating neuroinflammation and apoptosis after SCI requires further investigations (Bertero et al., 2020; McCully et al., 2020).

## Conclusion

We illustrated the therapeutic effects of transplantation of allogeneic mitochondria on traumatic SCI-induced somatosensory and functional impairments in rats. By determining the spatiotemporal distribution of viable transplanted mitochondria, we demonstrated the long-term survival of allogeneic mitochondria in the injured spinal cord of rats. Moreover, repressed mitochondrial fragmentation, apoptosis, oxidative stress, and inflammation may be likely involved in the mechanisms underlying the therapeutic effects of MT on SCI. Our findings provide basic evidence for the further translational application of MT in traumatic SCI. However, more detailed time-course studies on neuropathological changes after MT are needed, including the temporal expression of various pro-inflammatory cytokines and proteins associated with oxidative stress particularly at later stages of the injury, to better define the long-term therapeutic effects of MT on wound healing and functional improvements at the chronic stage after SCI.

## Data Availability Statement

The original contributions presented in the study are included in the article/Supplementary Material, further inquiries can be directed to the corresponding author.

## Ethics Statement

The animal study was reviewed and approved by the National Cheng Kung University Institutional Animal Care and Use Committee (IACUC approval number: 107184).

## Author Contributions

M-WL, S-YF, C-FL, and J-SL: study design. S-YF, J-SL, C-YH, P-HL, and H-FC: conducting animal experiments and measurement of the motor function. M-WL, H-FC, and J-SL: conducting tissue collection and analysis. S-YF, J-YH, and J-SL: statistical analysis and data interpretation. M-WL, J-YH, C-FL, and J-SL: manuscript preparation. All authors contributed to the article and approved the submitted version.

## Conflict of Interest

The authors declare that the research was conducted in the absence of any commercial or financial relationships that could be construed as a potential conflict of interest.

## Publisher’s Note

All claims expressed in this article are solely those of the authors and do not necessarily represent those of their affiliated organizations, or those of the publisher, the editors and the reviewers. Any product that may be evaluated in this article, or claim that may be made by its manufacturer, is not guaranteed or endorsed by the publisher.
