# Supplementary material for: Mitochondrial Transplantation Attenuates Neural Damage and Improves Locomotor Function After Traumatic Spinal Cord Injury in Rats
Source: Front Neurosci. 2022 Apr 12;16:800883. doi: 10.3389/fnins.2022.800883 (PMC9039257; doi:10.3389/fnins.2022.800883)
Supplement: Supplementary file 1 [file Data_Sheet_1.PDF]

Supplementary file

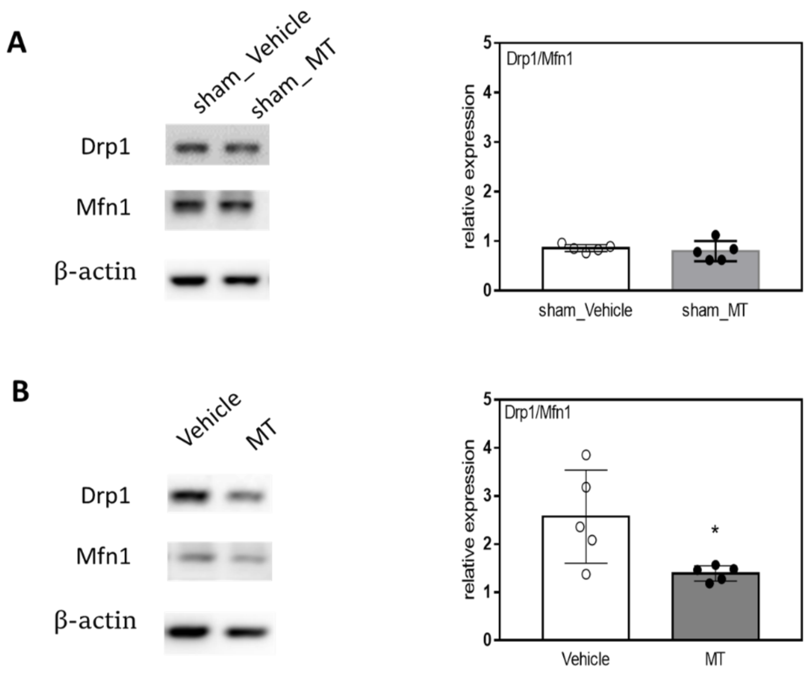

Figure 1. The ratio of Drp1 and Mfn1 protein expression levels in the sham (A) and SCI (B) groups.

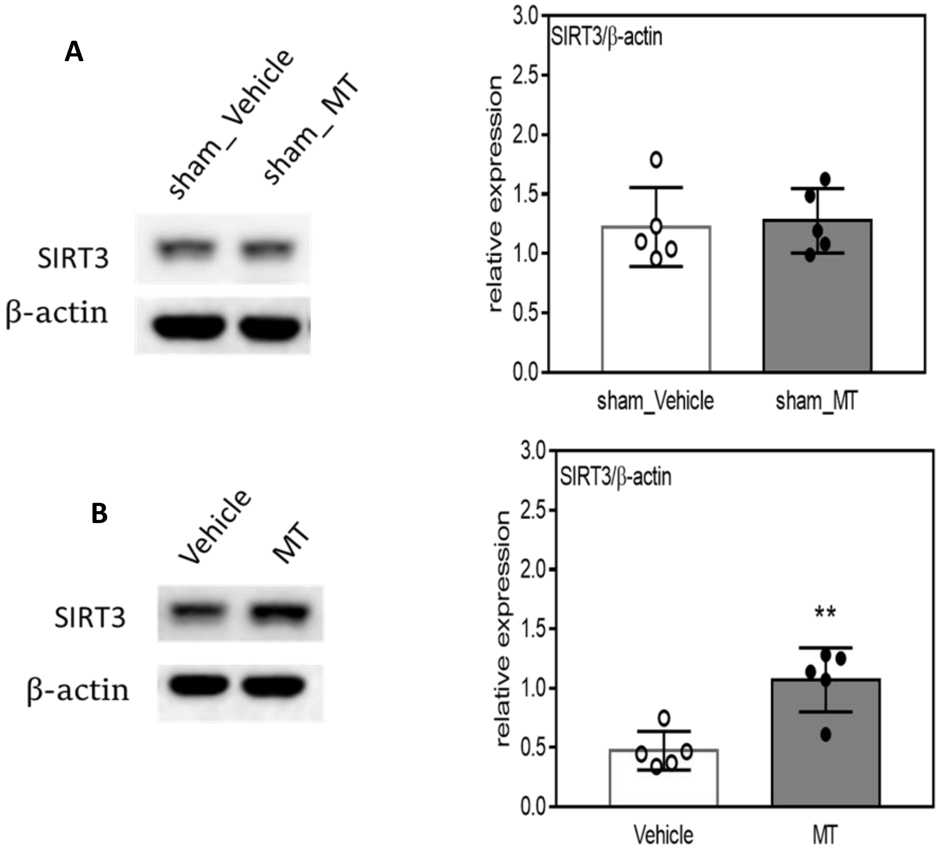

Figure 2. The protein expression of SIRT3 in the sham (A) and SCI (B) groups. SIRT3 protein was upregulated after MT in SCI groups.

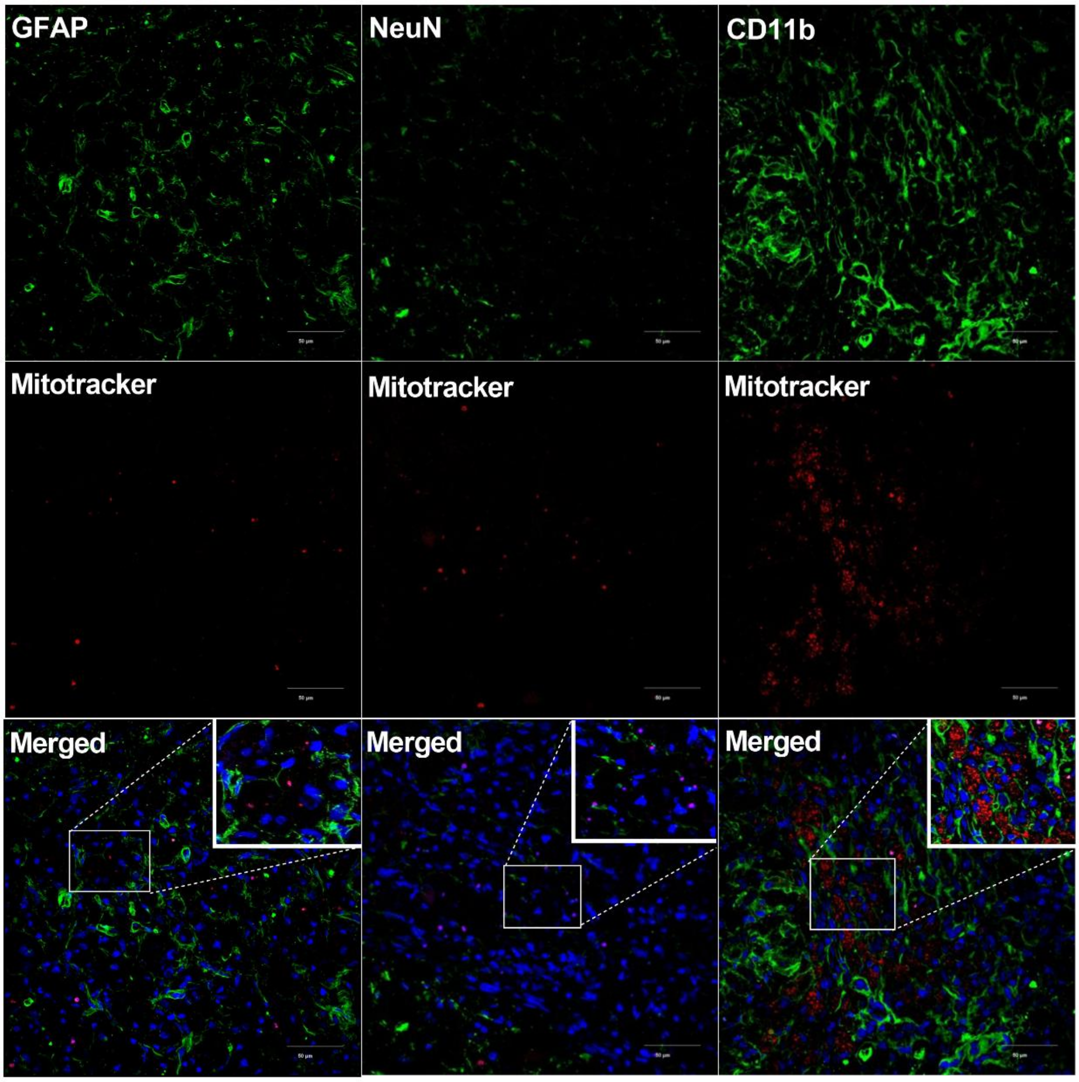

Figure 3. Colocalization of allogenic mitochondria with astrocyte, neuron, and microglia Co-staining GFAP (marker for astrocyte), NeuN (marker for neuron) and CD11b (marker for microglia) (green) with Mitotracker labeled mitochondria (red) and DAPI (blue). The magnified images show the transplanted mitochondria mainly distribute in the extracellular spaces. Scale bars: 50 μm
